# The Impact of the Blood Lipids Levels on Arterial Stiffness

**DOI:** 10.3390/jcdd10030127

**Published:** 2023-03-16

**Authors:** Mirela Baba, Mihaela Maris, Daniela Jianu, Constantin Tudor Luca, Dana Stoian, Ioana Mozos

**Affiliations:** 1 Center for Translational Research and Systems Medicine, “Victor Babeş” University of Medicine and Pharmacy, 300173 Timisoara, Romania; 2 Department of Functional Sciences-Pathophysiology, “Victor Babeş” University of Medicine and Pharmacy, 300173 Timisoara, Romania; 3 1st Department of Internal Medicine, “Victor Babeş” University of Medicine and Pharmacy, 300041 Timisoara, Romania; 4 Department of Internal Medicine, Military Hospital, 300080 Timisoara, Romania; 5 Department of Cardiology, “Victor Babeş” University of Medicine and Pharmacy, 300041 Timisoara, Romania; 6 Department of Cardiology, Institute of Cardiovascular Diseases, 300310 Timisoara, Romania; 7 2nd Department of Internal Medicine, “Victor Babeş” University of Medicine and Pharmacy, 300041 Timisoara, Romania; 8 Center of Molecular Research in Nephrology and Vascular Disease, “Victor Babeş” University of Medicine and Pharmacy, 300041 Timisoara, Romania

**Keywords:** arterial stiffness, vascular aging, blood lipids, atherogenic dyslipidemia, residual cardio-vascular risk

## Abstract

Arterial stiffness is a recognized predictor of cardiovascular morbidity and death. It is an early indicator of arteriosclerosis and is influenced by numerous risk factors and biological processes. The lipid metabolism is crucial and standard blood lipids, non-conventional lipid markers and lipid ratios are associated with arterial stiffness. The objective of this review was to determine which lipid metabolism marker has a greater correlation with vascular aging and arterial stiffness. Triglycerides (TG) are the standard blood lipids that have the strongest associations with arterial stiffness, and are often linked to the early stages of cardiovascular diseases, particularly in patients with low LDL-C levels. Studies often show that lipid ratios perform better overall than any of the individual variables used alone. The relation between arterial stiffness and TG/HDL-C has the strongest evidence. It is the lipid profile of atherogenic dyslipidemia that is found in several chronic cardio-metabolic disorders, and is considered one of the main causes of lipid-dependent residual risk, regardless of LDL-C concentration. Recently, the use of alternative lipid parameters has also been increasing. Both non-HDL and ApoB are very well correlated with arterial stiffness. Remnant cholesterol is also a promising alternative lipid parameter. The findings of this review suggest that the main focus should be on blood lipids and arterial stiffness, especially in individuals with cardio-metabolic disorders and residual cardiovascular risk.

## 1. Introduction

**Figure 1:**
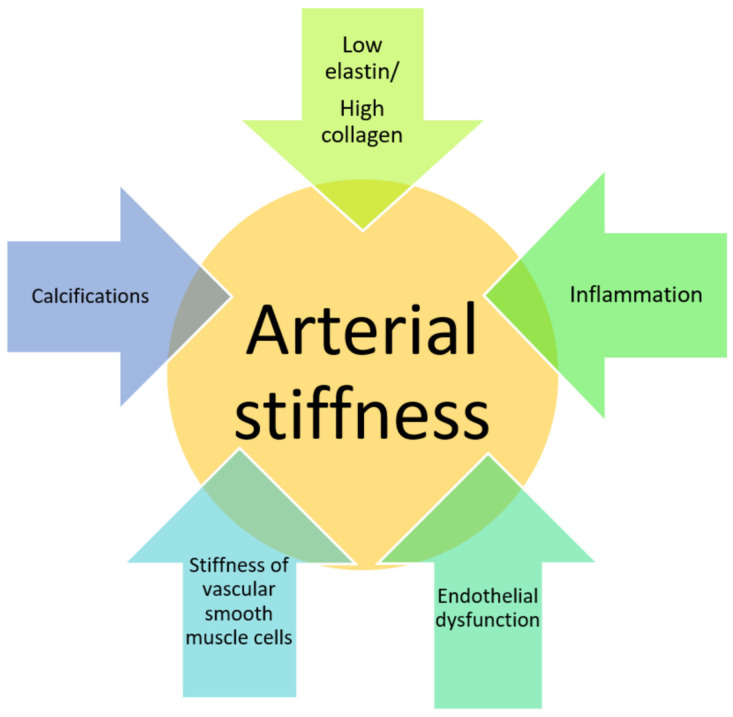
Factors that contribute to arterial wall stiffening.

Early vascular aging (EVA) is a concept that refers to premature alterations in arterial structure and function. The major change in EVA is increased arterial stiffness. Common factors that contribute to arterial wall stiffening include an impaired elastin/collagen ratio, inflammation brought on by reactive oxygen species, vascular calcifications, the stiffening of vascular smooth muscle cells and endothelial dysfunction (Figure 1) [1]. Arterial stiffness is a recognized predictor of cardiovascular (CV) morbidity and death, and is an early indicator of arteriosclerosis. Pulse wave velocity (PWV) is the most accessible, non-invasive and reproducible way to assess arterial stiffness. The main principle of PWV evaluation is that the pulse wave propagates down the artery tree at a velocity that is dependent on the elasticity of the wall itself; the stiffer the wall, the higher the propagation velocity. PWV is a simple indicator of the stiffness of medium and large arteries, due to the fact that stiffness generally occurs within elastic vessels, such as the aorta, as compared to muscular and resistive ones. Several methods, such as the carotid-femoral PWV (cf-PWV), brachial-ankle PWV (ba-PWV), and cardio ankle vascular index (CAVI), can be used to measure the stiffness of the arteries. There are several differences between these methods. The major difference refers to the non-aortic vessel used in the measurements. Aortic stiffness is evaluated using the cf-PWV, while assessments using the ba-PWV and CAVI methods include the use of medium-sized and resistive arteries. The techniques are not interchangeable, and the evidence supports the use of cf-PWV as the true gold standard for assessing arterial stiffness [2]. According to studies, an increased PWV predicts cardiovascular events independently of conventional cardiovascular risk variables [3,4,5], and may even greatly improve risk prediction in models that incorporate conventional risk factors.

The main feature of arterial stiffness, specifically in the large arteries, is arteriosclerosis, which is primarily caused by structural and functional changes in the vascular media. The thickening and hardening of the arterial wall, due to the replacement of elastic fibers with collagen, the destruction of muscle fibers and the presence of calcium deposits, with loss of the viscoelastic properties of the arteries, accompanies aging [6,7,8]. Atherosclerosis, a disease of the intima, is triggered by endothelial dysfunction, causing a narrowing of the arterial lumen, and arteriosclerosis share some risk factors, especially aging, nitrosative and oxidative stress, hypertension and inflammation; they are also both systemic diseases, as well as potentially having a parallel progression and a bi-directional relationship [8,9]. However, the two conditions are separate, and should not be confused for one another. Large artery stiffness significantly influences the development of cardiovascular diseases [2,10]. The arteries’ ability to absorb the cyclic variations in blood pressure, caused by intermittent left ventricular ejection, is reduced as they become stiffer. These reduced elasticities of the large arteries result in high systolic blood pressure and increased left ventricular afterload. Parallel to this, diastolic pressure decreases, impairing myocardial perfusion and increasing pulsatility, and damaging capillaries in high-flow organs, including the kidney and brain [11]. While it has long been believed that arterial stiffness only occurs as a result of organ damage caused by hypertension, more recent research indicates that arterial stiffness actually occurs before hypertension [12,13].

**Figure 2:**
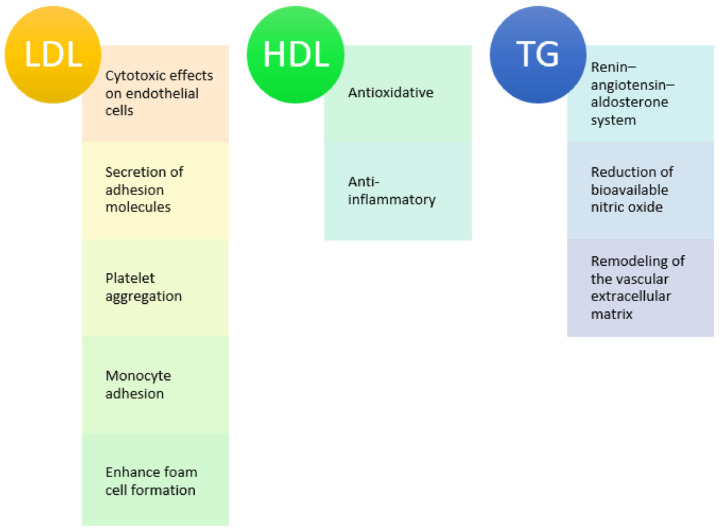
Effects of lipid markers on vascular function.

Arterial stiffness is influenced by numerous risk factors and biological processes. Arterial stiffness begins in early childhood, progresses in adulthood, and reflects the cumulative effects of cardiovascular risk factors on the vascular wall [14,15]. The occurrence and progression of arterial stiffness are closely linked to lipid and glucose metabolism, as well as to insulin resistance. Lipid metabolism is crucial, and standard blood lipids, non-conventional lipid markers and lipid ratios are associated with arterial stiffness [16]. There are some plausible explanations for the substantial correlation between the lipid profile and PWV [17] (Figure 2). Cholesterol binds to the arterial intima and accumulates in the arterial wall [18]. Arterial stiffness is accelerated by oxidative and nitrosative stress, caused by an excess of lipids [19]. Persistent inflammation in the arterial wall may be linked to lipids and the development of arterial stiffness. Lipids induce the production of cytokines and adhesion molecules from leukocytes, which in turn enable leukocytes to adhere to the vascular endothelium and penetrate the intima, increasing vascular resistance. In vitro cholesterol exposure causes detrimental modifications of elastin [20]. Biochemical tests have shown that lipids are linked to elastin, even in the early phases of atherosclerosis.

The objective of this review was to determine the lipid metabolism biomarker that is best correlated with vascular aging and arterial stiffness.

## 2. Standard Blood Lipids

### 2.1. TC (Total Cholesterol)

There are five major lipoproteins in the blood: chylomicrons, very-low-density lipoproteins (VLDL), intermediate-density lipoproteins (IDL), low-density lipoproteins (LDL) and high-density lipoproteins (HDL). The three main lipoprotein classes—VLDL, LDL and HDL—make up the majority of the TC distribution in humans. Measurement of plasma TC is needed to calculate the 10-year risk of fatal cardiovascular disease (CVD), as assessed by the SCORE chart [21]. According to the recent guidelines of the European Society of Cardiology on cardiovascular disease prevention in clinical practice, non-HDL cholesterol, including all atherogenic (Apo-B-containing) lipoproteins, is used as an input in the Systemic Coronary Risk Estimation 2 (SCORE2) and SCORE2-Older Persons (SCORE2-OP) risk algorithms [22].

Various studies, including subjects from different ethnic groups and different ages, have shown the relation between arterial stiffness and TC levels [17,23,24,25,26]. Participants with high brachial ankle pulse wave velocities (ba-PWV) had increased plasma levels of TC [24], and PWV is significantly correlated with a high TC [17,23,25,26].

### 2.2. LDL-C (Low Density Lipoprotein Cholesterol)

The retention of low-density lipoprotein cholesterol (LDL-C) and other cholesterol-rich apolipoprotein (Apo) B-containing lipoproteins within the arterial wall is the initiating event in atherogenesis. Plasma LDL-C should be measured, because LDL plays a causal role in the development of atherosclerotic cardiovascular disease (ASCVD) [21,22,27].

Plasma LDL-C can be measured directly, or it can be calculated using the Friedewald formula: LDL-C = TC − HDL-C − (TG/5) in mg/dL. The calculated LDL-C and direct LDL-C show very strong correlations. However, it has been discovered that the calculated LDL-C underestimates LDL-C levels at triglyceride (TG) concentrations greater than 177 mg/dL. With TG values of >400 mg/dL, the formula cannot be used. The calculated LDL-C may also be inaccurate at very low LDL-C levels, particularly when TG levels are high.

Damage to the arterial endothelium triggers a chronic inflammatory process known as atherosclerosis, which results in the remodeling of the arterial wall. Because oxidized LDL molecules trigger a variety of biological responses in macrophages, endothelial cells, T-cells and smooth muscle cells that encourage inflammation and atherogenesis, serum LDL-C plays a substantial role in this pathology.

The relation between PWV and LDL is unclear. Kilic et al. [23] found that in the group with the highest PWV, patients had higher LDL-C levels, but Wen et al. [28] found no significant association between high ba-PWV and elevated LDL. In a study conducted by Zhao et al. [25], LDL-C levels were significantly associated with ba-PWV, but the correlation of LDL-C with ba-PWV disappeared in female patients. Mozos et al. [29] reported significant correlations between LDL cholesterol levels and central systolic and diastolic blood pressure, PWV values and pulse pressure amplification (the ratio between central and peripheral pulse pressure) in a study including 56 patients, aged 48 ± 6 years, 57% males, with hypertension and high normal blood pressure. In a longitudinal prospective study [30], only LDL-C baseline levels were significantly associated with elevated carotid femoral PWV (cfPWV), while Zhan et al. [31] concluded that LDL levels were not significantly associated with ba-PWV. In a study [17] with a total of 909 participants aged 24 to 84 years, subjects had significantly higher LDL levels in the highest ba-PWV quartile, but the significance of the associations between LDL and ba-PWV disappeared after adjusting for age, body mass index (BMI) and smoking status. In another study [24], participants with high ba-PWV had increased plasma levels of LDL, but LDL-C was not able to predict arterial stiffness independently, after relevant confounders were taken into account. It is possible that other risk factors, such as age, as well as the presence of hypertension and diabetes, are masking the impact of LDL-C on arterial stiffness.

Patients with severe lipid abnormalities and/or a family history can be diagnosed as having genetic dyslipidemias. In therapy-naive patients, an LDL-C > 190 mg/dL requires cautious examination for potential familial hypercholesterolemia (FH) [21,22]. However, in those with lower LDL-C levels, potential FH should be taken into account if there is the presence of early atherosclerotic cardiovascular disease (ASCVD), or a family history of CVD. The application of the Dutch Clinical Lipid Network criteria is advised in addition to genetic testing, which is not always accessible, in order to identify a potential FH [21,22]. Patients who have familial hypercholesterolemia are at an extremely high risk for heart disease [32]. However, a meta-analysis of eight studies, involving 317 patients with FH and 244 non-FH individuals, suggested that FH patients do not have significantly altered PWV when compared with normocholesterolemic individuals [33]. In order to clarify the effect of FH on vascular stiffness, as evaluated by PWV, larger studies comparing PWV in FH patients with controls are certainly required [32].

### 2.3. HDL-C (High Density Lipoprotein Cholesterol)

HDL cholesterol plays an important role in removing cholesterol from atherosclerotic plaques and transporting it back to the liver. Low HDL cholesterol levels are the most prevalent lipid abnormalities in patients with early cardiovascular disease. Measuring HDL-C levels is advised, in order to further improve risk estimation, utilizing the online SCORE system, HeartScore (http://www.heartscore.org/en_GB/, accessed on 6 February 2023) [21]. Being involved in reverse cholesterol transport, HDL has additional atheroprotective effects, such as anti-inflammatory and antioxidative properties [34,35]. Aerobic physical activity, weight reduction and smoking cessation increase HDL-C levels [36,37]. However, randomized trials have not provided any evidence that increasing plasma HDL-C lowers the incidence of CV events [38]. Clinicians should be aware that there appears to be an elevated risk of ASCVD in those with extremely high HDL-C values (90 mg/dL); hence, at such levels, HDL-C cannot be used as a risk predictor [21,22].

The inverse relation between plasma HDL-C and the risk of ASCVD is reliable. In a study including Chinese hypertensive patients, HDL-C was negatively related to ba-PWV, TC and TG, even after adjusting for age, sex, BMI, and other cardiovascular risk factors [31]. In another study, low HDL levels were also significantly correlated with a high ba-PWV in the middle-aged and elderly Chinese population [25]. A high ba-PWV was also correlated with low HDL levels in healthy Japanese adolescents [26]. The structure and function of the arterial wall have been reported to be affected by atherosclerotic risk factors as early as the second decade of life [39]. Early detection of the loss of arterial wall elasticity offers crucial information on the development of subclinical vascular injury.

According to twin study heritability estimates, 62–77% of the variation in HDL cholesterol levels can be linked to genetic factors [40]. Apo AI and Apo AV are small proteins, primarily found as monomers on chylomicrons, VLDL, and HDL. In familial studies, the deletion of Apo AI has been linked to severe HDL deficits, and substantially accelerated coronary atherosclerosis [41]. Apolipoprotein A5 (*APOA5*) gene variations and hypertriglyceridemia have been linked in numerous studies; however, the relation between *APOA5* variants and HDL cholesterol levels has received less attention. A study from Korea searched for associations between *APOA5* genetic variants and arterial stiffness, in subjects with low HDL levels [40]. They revealed that low HDL cholesterol levels predisposed subjects with the *APOA5* rs662799 CC genotype to increased arterial stiffness. Lower levels of APOA I and APOA V, as well as a smaller LDL particle size, were found in individuals with the rs662799 CC genotype. The low HDL cholesterol group was slightly older, had a higher proportion of females and had a higher BMI than the control group. It also showed higher levels of triglycerides, glucose and insulin, even after adjustment for age, sex and BMI.

### 2.4. TG (Triglicerides)

TG analysis is recommended as part of the standard lipid analysis. The main carriers of plasma TGs in the blood are the chylomicrons, transporting dietary triglycerides from the intestine, and very low-density lipoproteins (VLDL), transporting triglycerides synthesized in the liver. Lipoprotein lipase is the main enzyme involved in the breakdown of triglycerides.

High TG levels were associated with an elevated ba-PWV [25,31], and they were even independent predictors of ba-PWV [24,26]. In a retrospective clinical research, TG levels were positively correlated with ba-PWV, even after adjusting for age, BMI, smoking status, fasting blood glucose (FBG), aspartate aminotransferase (AST), alanine aminotransferase (ALT) and estimated glomerular filtration rate (eGFR) [17]. Concluding, authors suggested that TGs could serve as a surrogate lipid marker for arterial stiffness.

The correlation between TG levels and PWV was also found in longitudinal prospective studies. Lower follow-up TG levels were significantly correlated with decrease carotid-femoral PWV, suggesting that lowering the TG levels may be an additional therapeutic strategy in patients with atherosclerotic disease [30]. In another follow-up study, even after adjusting for other risk factors, baseline TG was still able to predict the progression of arterial stiffness [42].

Serum triglyceride (TG) levels are yet to be fully understood in terms of their independent role as a cardiovascular risk factor. In the era of statin therapy, even after patients’ LDL-C goals have been achieved, we still need to be aware of the residual cardiovascular risk. Wen et al. [28] found that elevated TG levels presented the strongest correlation with high ba-PWV values, especially among participants with very low LDL-C (<70 mg/dL) levels, when compared with other groups’ LDL levels. Another cross-sectional study [43] had similar conclusions, and showed that as the LDL-C levels decreased, the proportion of high ba-PWV measurements in subjects with high TG levels gradually increased. The authors hypothesized that high TG levels are more closely linked to the early stages of CVD, particularly in patients with low LDL levels.

The final level of plasma TG is the cumulative effect of each person’s genetic predisposition, dietary choices (including the detrimental effects of increasing dietary fat and alcohol intake) and physical activity level (more physical activity lowers plasma TG levels) [44]. The percentage of the total plasma TG levels that is genetically determined is still unclear. Depending on the type of study, heritability estimates range from 40% to nearly 60% [45].

The apolipoprotein A5 gene (*APOA5*) is a key regulator of triglyceride levels [46]. Apolipoprotein A5 (*APOA5*) is a small protein located on triglyceride-rich lipoprotein particles (chylomicrons and VLDL) and on HDL. The effect of *APOA5* on plasma triglyceride levels has been demonstrated in research on both human and animal subjects. *APOA5* acts as an activator of lipoprotein lipase, the key enzyme in the breakdown of triglycerides [44].

**Figure 3:**
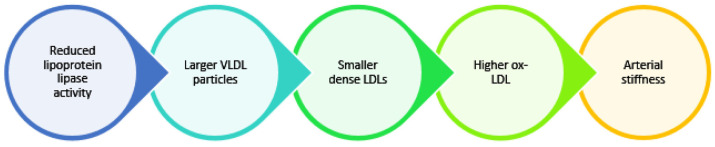
VLDLs and arterial stiffness (VLDL: very low-density lipoprotein, LDL: low-density lipoprotein, ox-LDL: oxidized-low-density lipoprotein).

As demonstrated in the study by Grosskopf et al. [47], *APOA5*-deficient mice showed reduced lipoprotein lipase activity and an accumulation of larger VLDL particles, which are precursors of small, dense LDLs. Carriers of a rare allele of the *APOA5* -1131T>C polymorphism have large VLDLs and small LDLs, according to a study by Guardiola et al. [48]. Small dense LDLs are more susceptible to in vitro oxidation. In a Korean case–control study [46], the -1131T>C polymorphism directly affected *APOA5* concentrations. Additionally, patients with hypertriglyceridemia who carried the *APOA5* -1131T>C mutation showed smaller LDL particle sizes, as well as higher ox-LDL and higher ba-PWV values. Therefore, the polymorphism *APOA5* -1131T>C makes hyper triglyceridemic patients more likely to have elevated atherogenic LDL levels, as well as increased arterial stiffness (Figure 3).

In the same study, malondialdehyde (MDA), which is the end product of lipid per-oxidation and a reliable clinical marker of oxidative stress, was positively correlated with baPWV. Serum MDA-LDL, which generates ox-LDL, has been shown to have direct cytotoxic effects on endothelial cells, stimulate the secretion of adhesion molecules, promote platelet aggregation and monocyte adhesion, and to enhance foam cell formation in atherosclerotic lesions, all of which have an impact on the baPWV, and result in the remodeling of vessel walls. In another study [49], high serum MDA-LDL levels in hemodialysis patients were positively correlated with cf-PWV values, and were significantly predictive of the appearance of high aortic stiffness.

The interaction between the influence of genetic and environmental factors on TG levels and the progression of arterial stiffness was studied in a 3-year prospective cohort study [50]. The key conclusion of this study was that, in healthy persons, the interaction between the *APOA5*-1131C variation and obesity can accelerate the age-related elevation of arterial stiffness, through controlling circulating triglyceride levels.

Angiopoietin-like protein 3 (ANGPTL3) is a secretory protein known to inhibit lipoprotein lipase, the enzyme that degrades circulating triglycerides in the capillaries of muscle and adipose tissue, and its inhibition leads to hypertriglyceridemia, which enables atherosclerotic plaque formation [51]. In a study in individuals with ANGPTL3 loss-of-function mutations, low total plasma levels of low-density lipoprotein cholesterol (LDL-C), low plasma levels of high-density lipoprotein cholesterol (HDL-C) and low plasma levels of triglycerides were found [52]. In another study [51], after multivariable adjustment, serum ANGPTL3 levels and an older age were found to be independently correlated with arterial stiffness in individuals with coronary artery disease (defined by >50% stenosis in any segment by coronary angiography).

**Figure 4:**
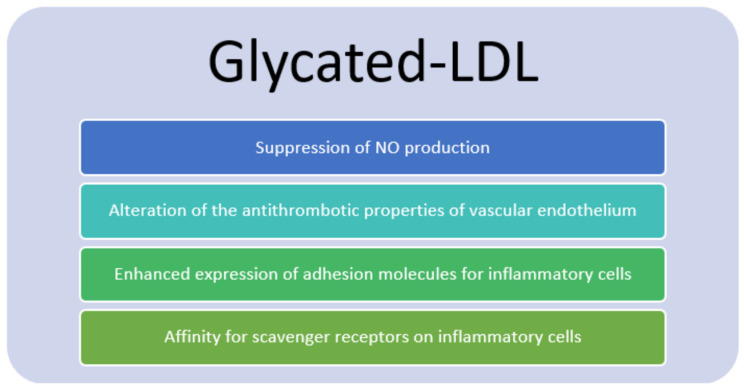
Proprieties of Glycated LDL (Glycated LDL: Glycated low-density lipoprotein, NO: nitric oxide).

Eating behavior can also influence the TG levels. Dietary carbohydrates are linked to hyperlipidemia and elevated cardiovascular risk [18]. Consuming more carbohydrates enables the liver’s de novo lipogenesis, which contributes to hypertriglyceridemia. The disposal of dietary fats is also altered by the simultaneous consumption of carbohydrates and lipids. It results in higher postprandial VLDL and chylomicron levels. In hyperglycemia, glucose spontaneously binds to proteins. Lipoproteins are modified by the non-enzymatic glycation of apolipoproteins. The attached sugar groups go through further alterations that result in the production of toxic advanced glycation end products (AGEs). Hyperglycemia contributes to hyperlipidemia due to the suppression of lipoprotein lipase. A reduction in post-prandial plasma lipoprotein lipase activity is correlated with the glycation of VLDL [53]. The diabetic pattern of dyslipidemia is associated with high concentrations of TG-rich lipoproteins [18]. The reduction of inflammation, brought on by better blood sugar control, may be mediated by the reduced glycation of lipoproteins. Glycated LDL has also toxic activities, such as the suppression of nitric oxide (NO) production [52], alteration of the anti-thrombotic properties of the vascular endothelium [54] and enhanced expression of adhesion molecules for inflammatory cells [55]. The uptake of lipoproteins through the conventional LDL receptor is also decreased by glycation [18]. When compared to traditional LDL receptors, which have a reduced affinity for glycated LDL, scavenger receptors on inflammatory cells are more effective at taking up glycated or oxidized lipoproteins (Figure 4).

## 3. Lipid Ratios

When comparing lipid parameters in terms of their strength of association with high PWV, studies often show that lipid ratios perform better overall than any of the individual variables used alone.

### 3.1. AIP—Atherogenic Index of Plasma: Log TG/HDL-C

Atherogenic dyslipidemia is a frequent lipid disorder linked to an elevated risk of cardiovascular disease (CVD) [56]. The lipid profile of atherogenic dyslipidemia is characterized by high levels of circulating triglycerides (TG) and low concentrations of high-density lipoprotein cholesterol (HDL-C). It is considered one of the main causes of lipid-dependent residual risk, regardless of LDL-C concentrations. Atherogenic dyslipidemia is present in several chronic cardio-metabolic disorders, such as pre-diabetes and diabetes, overweightness and obesity, metabolic syndrome and renal failure [56].

The relationship between arterial stiffness and TG/HDL-C was revealed by various studies. An elevated TG/HDL ratio was associated with high ba-PWV [57], and patients with arterial stiffness had higher TG/HDL ratios [56]. In a study with a total of 1015 participants, aged 18 to 44 years, without serious comorbidities, the presence of a high ba-PWV was significantly and independently predicted by the TG/HDL ratio [24]. From all of the conventional lipids and lipoprotein ratios, TG/HDL had the strongest association with the high ba-PWV. In a median 4.4 years follow-up study, including 659 apparently healthy men at high risk of arterial stiffness progression, both isolated TG levels and the TG/HDL-C ratio were able to predict arterial stiffness progression, even after adjustment for other risk factors [42]. TG/HDL was also significantly and independently related with arterial stiffness in normotensive and never-treated hypertensive subjects [58]. The authors suggested that TG/HDL should be included in the normal evaluations of clinical cardiovascular disease risk. In a retrospective observational study about the role of dyslipidemia in early vascular aging, TG/HDL-C levels were shown to be independent risk factors of increased PWV, and it was also suggested that the TG/HDL-C ratio might be used routinely for the prediction of early vascular aging and subclinical atherosclerosis [23]. In another prospective cohort study, including a non-normotensive population, followed up for approximately 4.71 years, the TG/HDL-C ratio was associated with a higher risk of arterial stiffness progression in the hypertensive population [59]. Newer evidence about the effects of TGs and HDLs on ASCVD risk is emerging from studies. TG/HDL-C was also found to be significantly linked with the prognosis of coronary artery disease (defined as stenosis >50%), coronary heart disease incidence, cardiovascular death, and overall mortality [60].

The correlation between TG/HDL and arterial stiffness may be explained by a number of different mechanisms. According to numerous studies, the plasma concentrations of TG and HDL-C are independently associated with insulin-mediated glucose disposal [61]. It has been reported that the TG/HDL ratio is a reliable surrogate index of insulin resistance [59]. The TG/HDL-C ratio may also be a quick and easy technique to determine as to whether someone is insulin-resistant, and thus at an elevated risk for cardiometabolic disease [61]. Vascular stiffness increases may represent the link between insulin resistance and hyperinsulinemia CVD [62].

Nitric oxide and endothelin-1 production, two crucial regulators of endothelial function, are out of balance in insulin resistance states, and their levels are decreased and elevated, respectively [61]. Under physiological circumstances, insulin controls the homeostasis of glucose by increasing glucose disposal in tissues that are sensitive to it [62]. It also controls the delivery of nutrients by vasodilating small supply arteries. Particularly, insulin-mediated nitric oxide (NO) generation from the vascular endothelium increases blood flow, while improving glucose disposal. A reduction in the sensitivity or response to the metabolic effects of insulin is referred to as insulin resistance. In physiological conditions, vascular smooth muscle cells’ migration and proliferation, as well as excessive oxidative stress, inflammation, leukocyte adhesion, and intracellular migration, are inhibited by endothelial cell NO production. Reduced NO availability, in turn, causes vascular smooth muscle cells’ (VSMC) cytoskeletal remodeling and altered integrin-mediated adhesion to extracellular matrix proteins that promote intrinsic VSMC stiffening [63]. This, combined with the deposition of matrix proteins and fibrosis, causes arterial stiffness.

**Figure 5:**
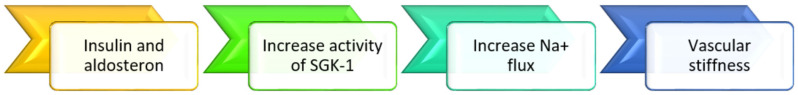
Insulin and aldosterone and the development of vascular stiffness (SGK-1: Serum and glucocorticoid kinase 1).

Moreover, increased insulin and aldosterone levels play a role in the development of vascular stiffness in insulin-resistant diseases. Insulin resistance and chronic hyperinsulinemia stimulate the expression of angiotensin II receptors in vascular tissue and local renin–angiotensin–aldosterone system activity, which results in vessel wall hypertrophy and fibrosis, decreasing arterial elasticity [61]. Both aldosterone and insulin increase the activity of serum and glucocorticoid kinase 1 (SGK-1), a key regulator of sodium (Na+) channel function in the blood vessels and the kidneys [62]. Observations indicating that gain-of-function mutations in SGK-1 in humans cause hypertension, insulin resistance, and obesity underscore the significance of SGK-1 in the pathophysiology. An increase in Na+ flux causes the cytoskeleton to reorganize, NO bioavailability to decline and increased vascular stiffness (Figure 5). Over a period of months, rats on a diet high in refined carbs and saturated fat developed insulin resistance. Increased endothelial cell (EC) Na+ channel activity was linked to the stiffening of the EC, aorta, and cardiomyocytes.

### 3.2. AI—Atherogenic Index: Non-HDL/HDL

Non-HDL is calculated as TC–HDL-C. In a study comparing the relationship between lipid profiles and lipid ratios, respectively, with arterial stiffness [25], participants with higher non-HDL-C/HDL-C ratios had a higher risk of arterial stiffness than patients with other lipid parameters. Since the non-HDL-C/HDL-C method is simple and affordable, the authors suggest considering non-HDL-C/HDL-C as a substitute indicator of arterial stiffness in clinical practice. Another study, including young men [24], showed that AI positively and significantly predicted the presence of elevated ba-PWV, even after adjustments for other risk factors, which was not the case for isolated non-HDL. According to another study [57], partial correlation analysis showed that ba-PWV was correlated with non-HDL/HDL in hypertensive patients.

### 3.3. Castelli Risk Index I: TC/HDL-C

According to different studies [23,25,57], participants with increased ba-PWV had higher TC/HDL concentrations. In another study, the presence of a high ba-PWV was significantly and independently predicted by Castelli risk index I [24]. The ratio of TC/HDL-C, but not the isolated TC level, was found to be independently and positively associated with ba-PWV, after adjustment for other non-lipid risk factors. In a cross-sectional study [28], the TC/HDL-C ratio was consistently associated with arterial stiffness across the range of LDL-C levels, even when they were below 70 mg/dL. This was the first study to report significant positive associations of the TC/HDL ratio with arterial stiffness, irrespective of the LDL-C level.

### 3.4. Castelli Risk Index II: LDL-C/HDL-C

LDL/HDL has a weaker correlation with PWV. In a Chinese middle-aged and elderly population, which is known to have a high prevalence of arterial stiffness [31], participants with high ba-PWV values showed higher levels of LDL/HDL. Moreover, LDL-C/HDL-C had a positive relation with the ba-PWV value. The levels of LDL/HDL were significantly higher in men than in women, but overall, the female population, with high ba-PWV values, had higher levels of LDL-C/HDL-C. Another study [23] showed that LDL/HDL levels were significantly higher in patients with high PWV values.

## 4. Alternative Lipid Parameters

As we all know, the blood lipid that has attracted the most attention is LDL-C, but a large number of studies have shown that many individuals with optimal LDL-C levels, or even significantly reduced LDL-C levels, still experience atherosclerosis progression and even cardiovascular events. This phenomenon is known as the residual risk, which cannot be identified by measuring LDL-C levels. Interest has been increasing in the use of alternative lipid parameters.

### 4.1. Non-HDL and ApoB

Non-HDL-C contains the cholesterol carried by all potentially atherogenic lipoprotein particles, including LDL-C, intermediate-density lipoproteins (IDL), very-low-density lipoproteins (VLDL) and lipoprotein a (LPa). Apolipoprotein B (ApoB) represents the number of atherogenic lipoprotein particles mentioned, because each lipoprotein particle contains one molecule of ApoB. ApoB is a distinct measurement from non-HDL-C, because it gives a precise estimate of the number of atherogenic particles in circulation, while non-HDL-C represents the mass of atherogenic cholesterol. Generally, there is a strong correlation between the concentrations of LDL-C, non-HDL-C and ApoB. As a result, they typically offer relatively comparable information regarding the risk of atherosclerotic cardiovascular disease (ASCVD). The estimated or directly measured LDL-C level, however, may underestimate the cardiovascular risk in some cases, such as among individuals with elevated TG levels, diabetes mellitus, obesity, or very-low LDL-C levels. Underestimating the total concentration of ApoB-containing lipoproteins means underestimating the risk of ASCVD in some cases. According to the recent ESC/ASA Guidelines for the management of dyslipidemia lipid modification to reduce cardiovascular risk, around 20% of patients may have discordance between measured LDL-C and ApoB levels [21]. In a paper about the discordance between apolipoprotein B, non-HDL cholesterol and LDL cholesterol levels in middle-aged and elderly Chinese patients, as well as their use to predict arterial stiffness, the results showed that LDL-C was discordant with non-HDL-C in a proportion of 20.1%, and was discordant with ApoB in a proportion of 30.8% [64]. Lower levels of non-HDL-C and ApoB were linked to lower ba-PWV, whereas higher levels of non-HDL-C and ApoB were linked to higher ba-PWV. When non-HDL-C or ApoB levels in the discordant groups were higher than the cut-off value, the probabilities for higher arterial stiffness were noticeably increased. To further improve arterial stiffness, lipid-lowering medications in these patients should additionally target non-HDL-C and ApoB levels, in addition to LDL-C levels.

In the presence of endothelial dysfunction, all ApoB-containing lipoproteins with a diameter of less than 70 nm, including smaller TG-rich lipoproteins and their remnant particles, can cross the endothelial barrier, where they may become trapped after interacting with extracellular structures, such as proteoglycans [65]. Retained ApoB-containing lipoproteins cause a complicated chain of events that results in lipid accumulation and the formation of an atheroma. Long-term exposure to ApoB-containing lipoproteins results in more particles accumulating over time in the arterial wall, and in the growth and progression of atherosclerotic plaques.

#### 4.1.1. Non-HDL

Non-HDL-C can be calculated as TC–HDL-C and is a measure of the TC carried by all atherogenic ApoB-containing lipoproteins. According to 2019 ESC/EAS Guidelines for the management of dyslipidemias, non-HDL-C evaluation has a recommendation class of level C for risk assessment, particularly in people with high TG levels, DM, obesity, or very-low LDL-C levels [21]. Non-HDL cholesterol, including all atherogenic (Apo-B-containing) lipoproteins, is used as an input in the Systemic Coronary Risk Estimation 2 (SCORE2) and SCORE2-Older Persons (SCORE2-OP) risk algorithms, according to the most recent European Society of Cardiology guidelines on cardiovascular disease prevention in clinical practice. For all patients, especially those with hypertriglyceridemia or diabetes, it is recommended as a reasonable alternative treatment goal [22].

There are many studies that attempt to evaluate the correlations between elevated non-HDL and arterial stiffness, cardiovascular risk and major adverse cardiovascular events. A strong correlation has been found, between high non-HDL levels and arterial stiffness, as assessed by PWV [23,24,25,31,66,67]. In one study on 1582 participants, mean age 52.8, patients in the highest PWV group had higher non-HDL levels, and these elevated non-HDL levels were an independent risk factor of increased PWV in logistic regression analysis [23]. The study conducted by Wen et al. reported a significant positive association of non-HDL-C with arterial stiffness, irrespective of LDL-C levels, especially among participants with naturally very-low LDL-C levels [28]. Even among individuals with LDL-C values of <70 mg/dL, elevated non-HDL-C was associated with high ba-PWV. Among participants with an LDL-C of 100–129.9 mg/dL, non-HDL-C was not an independent risk factors for high ba-PWV, but revealed a significant association. A prospective study, including 7276 participants without arterial stiffness at baseline, after a 1.78 years follow-up period, showed that the risk of incident arterial stiffness was significantly higher among younger participants with high non-HDL levels [68]. Puri et al. discovered that variations in non-HDL-C are strongly related to plaque progression [69]. Ito et al. analyzed 8383 people in the general Japanese population, and found that a one standard deviation increase in non-HDL-C was linked with a 37-fold greater risk of incident coronary artery disease (CAD) [70]. In a randomized controlled trial searching for major adverse cardiovascular events (MACEs) in a high-risk, primary prevention population of 6901 subjects, after a median follow-up of 4.8 years, the serum concentrations of non-HDL-C were associated with 5% higher risk of MACEs per every 10 mg/dL increase [56].

#### 4.1.2. Apo B

Apolipoprotein B (ApoB) analysis is recommended in risk assessments, particularly in people with high TG levels, DM, obesity, metabolic syndrome, or very-low LDL-C levels. It can be used as an alternative to LDL-C analysis, if available, as the primary measurement for screening, diagnosis, and management, and it may be preferred over non-HDL-C levels, in people with high TG levels, DM, obesity, or very-low LDL-C levels. For those at a very-high risk, with recurrent ASCVD events, a goal of ApoB < 55 mg/dL may be considered. When compared to measuring or calculating LDL-C and non-HDL-C levels, ApoB measurement methods are superior. Fasting is not necessary, since even in the post-prandial state, chylomicrons that contain ApoB48 normally make up less than 1% of the total amount of circulating ApoB-containing lipoproteins. The majority of Apo(B) is ApoB100.

According to a cross-sectional study, including 1517 individuals [67], increasing ApoB levels were associated with a higher prevalence of CVD in both men and women. In participants with ApoB levels in the top quartile, all stiffness parameters, including PWV, augmentation index (AIx), central augmented pressure (CAP), central systolic pressure (CSP), and central diastolic pressure (CDP), were elevated. In this study, both ApoB and non-HDL-C were significantly better than LDL-C in identifying people with an elevated CVD risk. LDL-C was shown to be the least effective in this population-based sample, and non-HDL-C was not as effective as ApoB. Therefore, these findings favor the atherogenic particle number (ApoB) over the total mass of atherogenic cholesterol (non-HDL-C). After adjustment for other major CV risk factors, including HDL-C, fasting glucose, waist circumference, pulse pressure and pack-years of smoking, the impact of ApoB on subclinical atherosclerosis declined. These findings are related to additional major CV risk factors involved in the development of subclinical atherosclerosis.

### 4.2. Lipoprotein (a)

Lp(a) is an LDL particle with an Apo(a) moiety covalently attached to its ApoB compo-nent [71]. It is <70 nm and can freely pass through the endothelial barrier, where it may be retained in the artery wall, similarly to LDL, increasing the risk of ASCVD [21]. Although higher plasma Lp(a) concentrations are linked to an increased risk of ASCVD, Lp(a) seems to be a much weaker risk factor than LDL-C for most people [72]. Because about 90% of a person’s Lp(a) level is inherited, every adult should have their Lp(a) levels measured at least once in their lifetime, in order to identify those who may have a very-high lifetime risk of ASCVD, due to very high inherited Lp(a) levels >180 mg/dL [73]. Lp(a) should be taken into consideration in certain patients who have a family history of early CVD, and for reclassification in people who are on the threshold of moderate and high risk [21]. Wide mechanistic studies have revealed proatherogenic, proinflammatory, and even prothrombotic effects, the latter due to its structural similarity to plasminogen [21]. Brosolo et al. [74] showed that plasma Lp(a) levels are significantly and directly related to markers of arterial stiffening in middle-aged patients with uncomplicated hypertension. For the brachial augmentation index (Aix), this connection is unaffected by confounders, indicating preferential effects on the peripheral arterial tree.

### 4.3. Remnant Cholesterol (RC)

Remnant cholesterol is the amount of cholesterol found in triglyceride-rich remnant lipoproteins, such as the very low-density lipoproteins (VLDLs) and intermediate-density lipoproteins (IDLs) that are present in the fasting state, as well as additional chylomicron remnants in the non-fasting state [75]. Remnant cholesterol can be measured or calculated as non-fasting total cholesterol minus HDL cholesterol minus LDL cholesterol; thus, when triglycerides are <72 mg/dL (<4.0 mmol/L), remnant cholesterol is calculated as triglycerides × 0.45 [75].

In individuals with normal TG, for every chylomicron remnant particle, there are approximately 10 VLDL particles that have a short half-life compared to LDL. Considering that for every VLDL particle there are approximately nine LDL particles, this proportion could explain why a rise in plasma TG will lead to many more LDL particles than VLDL particles [76]. Both human and animal studies have shown that triglyceride-rich lipoproteins can enter the arterial intima [77]. Most likely, lipoprotein lipase will degrade triglyceride-rich lipoproteins that are trapped in the arterial intima [78]. Furthermore, whereas LDL particles must first undergo modification, triglycerides can be taken up directly by macrophages, which promotes a faster production of foam cells [79]. High levels of remnant-C in serum would contribute to an increased penetration into the artery wall.

In the high-risk primary prevention study from Spain, remnant-C was associated with a 21% higher risk of major adverse cardiovascular events (MACE) per 10 mg/dL increase [56]. Men and women had comparable distributions of remnant-C, but there were differences according to BMI categories and diabetes status. A higher BMI, triglyceride levels, hypertension prevalence, reduced physical activity, and reduced HDL-C were all observed in those with elevated remnant-C. Regardless of LDL-C concentrations, subjects with a remnant-C > 30 mg/dL were recognized as having a higher cardiovascular risk, and, independent of LDL-C levels, MACE incidence was lowest in the low remnant-C groups. These findings demonstrated that in a cohort of high-risk Mediterranean subjects with a high prevalence of diabetes and obesity, levels of triglycerides and estimated remnant-C, but not LDL-C or HDL-C, were associated with CVD outcomes independently of lifestyle characteristics and other cardiovascular risk factors.

In an observational follow-up study, lower remnant cholesterol levels of 32 mg/dL and 81 mg/dL were revealed to reduce recurrent MACE by 20% and 50%, respectively, in patients with a diagnosis of myocardial infarction or ischemic stroke [77]. These findings point to the necessity of secondary prevention in people with elevated remnant cholesterol levels.

A study conducted by Wang et al. [66] was the first one to report the association between remnant cholesterol and arterial stiffness. Participants in the arterial stiffness group had noticeably higher remnant cholesterol than those in the non-arterial stiffness group. The results from this study showed that in the general population, free of cardiovascular illness, RC is independently related to the risk of arterial stiffness. In the comparative analysis of the area under the ROC curve (AUC) of RC and other non-conventional lipid parameters for predicting arterial stiffness, the discriminant ability of RC was significantly higher than that of other predictive models, including non-HDL-C, AIP, AI, TG/HDL-C, CRI-I and CRI-II. Another study with 8028 participants older than 40 years confirmed the strong and independent association of RC with ba-PWV [80].

## 5. Discussion and Further Research Directions

The most relevant studies are presented in tables that show correlations between lipid parameters or lipid ratios and arterial stiffness (Table 1 and Table 2). The correlations appear in both cross-sectional and longitudinal studies, but there are, unfortunately, significantly fewer longitudinal studies. Further prospective longitudinal studies are required in the future to establish more important associations between lipids and arterial stiffness over time. There is also a need for more cf-PWV studies, because most assessment techniques used tonometric or oscilometric ba-PWV.

**Figure 6:**
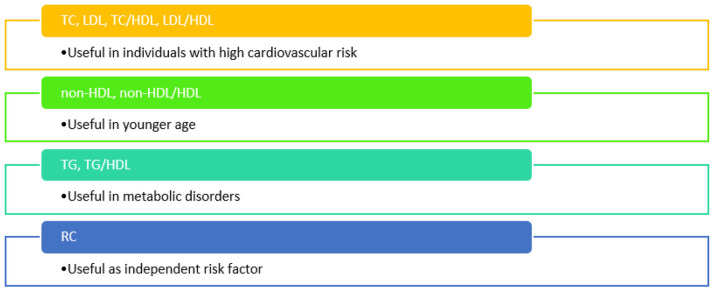
Lipid parameters and ratios associated with arterial stiffness in particular conditions (TC = total cholesterol, TG = triglycerides, LDL = low-density lipoprotein cholesterol, HDL = high-density lipoprotein cholesterol, non-HDL = non-high-density lipoprotein cholesterol, RC = remnant cholesterol).

Some of the lipid parameters and ratios were associated with arterial stiffness more often in particular conditions (Figure 6).

Different cardiovascular risk factors are associated with different blood lipid abnormalities and accelerate the increase of arterial stiffness. Age is almost always relevant in studies and is by far the most prevalent factor. Systolic blood pressure is a well-known factor associated with arterial stiffness, and has also a strong correlation with blood lipid levels [23,24,30,31,46,50,51,56,62,63,66,81]. Fasting blood glucose or established diabetes mellitus [17,23,24,30,51,62,63,79] and metabolic syndrome [26,81,82,83] have shown a significant association with elevated blood lipids and increased arterial stiffness.

Studying how different lipids affect arterial stiffness may identify parameters that can be targeted, in order to maintain healthier arteries. Lifestyle interventions and lipid-lowering therapies, including statins and PCSK9 inhibitors, are effective in reducing arterial stiffness in several patient groups [8,84,85,86,87,88].

## 6. Conclusions

Arterial stiffness is influenced by numerous risk factors and biological processes. The lipid metabolism biomarkers include standard blood lipids, non-conventional lipid markers and lipid ratios. Triglycerides (TG) are the standard blood lipids that have the strongest associations with arterial stiffness, and they are often linked to the early stages of cardiovascular diseases, particularly in patients with low LDL levels. When comparing lipid parameters in terms of their strength of association with high PWV, studies often show that lipid ratios perform better overall than any of the individual variables used alone. The relation between arterial stiffness and TG/HDL-C has the strongest evidence in various studies. It is the lipid profile of atherogenic dyslipidemia that is found in several chronic cardio-metabolic disorders, such as pre-diabetes, diabetes mellitus, overweightness, obesity and metabolic syndrome, and it is characterized by high circulating triglycerides (TG) and low concentrations of high-density lipoprotein cholesterol (HDL-C). It is considered to be one of the main causes of lipid-dependent residual risk, regardless of LDL-C concentration. Recently the use of alternative lipid parameters has also been increasing. Both non-HDL-C, which contains the cholesterol carried by all potentially atherogenic lipoprotein particles, and ApoB, which represents the number of atherogenic lipoprotein particles, are very well-correlated with arterial stiffness. Remnant cholesterol is a promising alternative lipid parameter that represents the amount of cholesterol found in triglyceride-rich remnant lipoproteins, and has a substantial correlation with cardiovascular risk, both in primary and secondary prevention. The relation with arterial stiffness has also been found and more prospective studies are needed. We suggest that the main focus on blood lipids and arterial stiffness should be especially in individuals with cardio-metabolic disorders and residual cardiovascular risk.

## Figures and Tables

**Table 1:** Most relevant studies with lipid parameters and arterial stiffness (ABI = ankle-branchial index, ABImax = maximal ABI, ApoB = plasma apolipoprotein, ba-PWV = brachial-ankle pulse wave velocity, cf-PWV = carotid-femoral PWV, FBG = Fasting blood glucose, HDL-C = high density lipoprotein cholesterol, LDL-C = low density lipoprotein cholesterol, PWV = pulse wave velocity, TC = fasting total cholesterol, TG = triglycerides, RC = remnant cholesterol, Scr = serum creatinine, UA = uric acid).

Study Participants	Methodology	Results	Conclusions	References
11,640 subjects, Japanese general population, stratified into 4 groups according to LDL-C level.	Serum levels of TG and LDL-C, ba-PWV.	As the LDL-C levels decreased, the proportion of high ba-PWV measurements in subjects with high TG levels gradually increased.	High TG levels in subjects with low LDL-C levels were significantly associated with increased arterial stiffness.	Kawasoe et al., 2018 [43]
1447 subjects.	Longitudinal prospective study, median follow-up 4.8 years TC, TG, HDL-C, LDL-C, cf-PWV and carotid-radial PWV.	TGs were independently associated with cf-PWV and carotid-radial PWV. In the group older than 65 years, the association between baseline TG levels and follow-up cf-PWV and carotid-radial PWV were strengthened.	Achieving low TG levels may be an additional therapeutic consideration in subjects with atherosclerotic disease.	Wang et al., 2016 [30]
909 subjects aged 24 to 84 years were stratified into four ba-PWV quartiles.	TC, TG, HDL, and LDL levels. ba-PWV and the ABImax (automatic waveform analyzer.	HDL levels were inversely associated with ba-PWV after adjustment for cardiovascular risks, and TG levels were positively related to ba-PWV independent of cardiovascular risks and liver function.	TGs may be a surrogate lipid marker of arterial stiffness.	Wang et al., 2020 [17]
16,733 participants urban Chinese residents.	Serum TG, TC, LDL-C, HDL-C levels. ba-PWV (noninvasive atherosclerosis measurement system).	Non-HDL-C, TG, and the TC/HDL-C ratio were consistently and positively associated with ba-PWV, independent of CVD risk factors. Positive associations were observed within every LDL-C level investigated, including LDL-C.	Associations of non-HDL-C, TG, and the TC/HDL-C ratio with arterial stiffness over a range of LDL-C concentrations, even those that are optimal.	Wen et al., 2019 [28]
14,071 participants, rural Chinese adults with primary hypertension.	Fasting TC, TG, HDL-C. ba-PWV (automatically measured using PWV/ABI instruments).	TC, TG, and non-HDL-C values were significantly associated with ba-PWV, and HDL-C. was negatively related to ba-PWV	Supports using non-HDL-C as a biochemical indicator in clinical settings.	Zhan et al., 2019 [31]
7276 participants without arterial stiffness at baseline.	Follow-up 1.78 years, TC, HDL-C, LDL-C, TG, FBG. Non-HDL-C was calculated as TC minus HDL-C, ba-PWV, ABI (automatic waveform analyzer).	The risk of incident arterial stiffness was significantly higher among younger participants with high non-HDL levels.	Non-HDL-C identified as a potential risk factor of arterial stiffness, especially for younger people.	Wang et al., 2022 [68]
402 middle-aged and elderly Northern Chinese individuals.	FBG, TC, TG, HDL-C, LDL-C, ApoB, Scr, UA and homocysteine (automatic biochemical analyzer), ba-PWV (oscillometer-based device).	There was discordance between LDL-C and non-HDL-C, and ApoB, in middle-aged and elderly Chinese individuals; this was associated with a higher risk of arterial stiffness.	Lipid-lowering therapy should not only focus on LDL-C levels, but also on non-HDL-C and ApoB levels to further reduce arterial stiffness.	Qu et al., 2021 [64]
912 participants aged 24–84 years	TC, TG, LDL-C, HDL-C, Non-conventional lipid parameters (calculated according to formulas). ba-PWV (automatic waveform analyzer from Colin Medical Technology).	Participants in the arterial stiffness group had significantly higher remnant cholesterol compared with the non-arterial stiffness group.	Remnant cholesterol is independently associated with the risk of arterial stiffness in the general population free from cardiovascular disease.	Wang et al., 2022 [66]
8028 participants.	RC, TG, TC, LDL-C, non-HDL-C, ba-PWV (Omron Colin BP-203RPE III device).	The concentrations of RC, TG, TC, LDL-C, and non-HDL-C were all positively and independently associated with ba-PWV. The HDL-C concentration was inversely associated with ba-PWV.	The RC and TG concentrations have stronger associations with arterial stiffness than other lipid indices.	Liu et al., 2022 [80]

**Table 2:** Most relevant studies with lipid ratios and arterial stiffness (ABI = ankle-brachial index, AI = atherogenic indices, AIP = atherogenic index of plasma, BP = blood pressure, DM = diabetes mellitus, TG = triglycerides, LDL-C = low-density lipoprotein cholesterol, HDL-C = high-density lipoprotein cholesterol, non-HDL-C = non-high-density lipoprotein cholesterol, ba-PWV = brachial-ankle pulse wave velocity).

Study Participants	Methodology	Results	Conclusions	References
380 subjects, grouped into four groups: Group A (no history of hypertension and dyslipidemia); Group B (a history of dyslipidemia but not hypertension); Group C (a history of hypertension but not dyslipidemia); and Group D (a history of both hypertension and dyslipidemia).	Complex lipid indices were calculated according to the TC, TG, LDL-C and HDL-C parameters. The ba-PWV was measured using a volume plethysmography device.	Hypertension and dyslipidemia were independent factors for ba-PWV; AI, AIP and TC/HDL-C correlated with ba-PWV.	Hypertension and/or dyslipidemia might represent risk factors for arterial stiffness; in patients with hypertension, TC, HDL-C, AIP, AI and TC/HDL-C might be correlated with arterial stiffness, and the pathological increase may be a risk factor for arterial stiffness.	Si et al., 2019 [57]
1582 participants.	FBG, TG, TC, HDL-C, LDL-C, plasma creatinine, office BP, ambulatory BP, PWV, and other arterial stiffness parameters (Mobil-O-Graph).	DM, age, non-HDL and TG/HDL-C levels were independent risk factors of increased PWV.	The TG/HDL-C ratio might be used routinely for the prediction of early vascular aging and subclinical atherosclerosis.	Kilic et al., 2021 [23]
615 participants with no lipid-lowering or BP-lowering medication.	TC, HDL-C, LDL-C, TG, continuous pulse wave and radial BP (tonometric sensor).	AIP was a significant independent explanatory factor for PWV.	The link between AIP and large arterial stiffness supports the view that the calculation of AIP should be included in the normal clinical cardiovascular disease risk evaluation.	Choudhary et al., 2019 [58]
1015 male participants aged 18 to 44 years without serious comorbidities.	TC, HDL-C, LDL-C, TG. ba-PWV (noninvasive vascular screening device).	The lipid parameter with the strongest association was TG/HDL-C.	Lipid ratios are superior to conventional lipid parameters for predicting arterial stiffness in young Chinese men. The TG/HDL-C ratio can be a potential target for intervention to reduce arterial stiffness in clinical practice	Wen et al., 2017 [24]
1133 participants, middle-aged and elderly Chinese.	TC, TG, HDL-C, LDL-C. ba-PWV (VP-1000 Automatic Arteriosclerosis Measurement System)	Individuals with higher non-HDL-C/HDL-C levels have a higher risk of arterial stiffness than other lipid parameters.	The non-HDL-C/HDL-C ratio can represent a surrogate indicator of arterial stiffness in clinical practice.	Zhao et al., 2014 [25]
659 healthy males; subjects were classified into two subgroups: those who decreased their quartile distribution or persisted within the two lower quartile groups, and those who increased their quartile distribution or persisted within the two higher quartile groups.	Follow up, 4.4 years. TC, TG, LDL-C, HDL-C, ba-PWV and ABI.	Baseline TG and TG/HDL-C were able to predict arterial stiffness progression even after adjustment for other risk factors.	Positive association between TG, TG/HDL-C, and PWV progression should be considered in the management of vascular health.	Sang et al., 2021 [42]

## Data Availability

Not applicable.
